# Waste Mismanagement in Developing Countries: A Review of Global Issues

**DOI:** 10.3390/ijerph16061060

**Published:** 2019-03-24

**Authors:** Navarro Ferronato, Vincenzo Torretta

**Affiliations:** Department of Theoretical and Applied Sciences, University of Insubria, Via G.B. Vico 46, I-21100 Varese, Italy; vincenzo.torretta@uninsubria.it

**Keywords:** environmental contamination, public health, solid waste management, sustainability, open dumping, informal recycling, open burning, sustainable development, hazardous waste, risk assessment

## Abstract

Environmental contamination due to solid waste mismanagement is a global issue. Open dumping and open burning are the main implemented waste treatment and final disposal systems, mainly visible in low-income countries. This paper reviews the main impacts due to waste mismanagement in developing countries, focusing on environmental contamination and social issues. The activity of the informal sector in developing cities was also reviewed, focusing on the main health risks due to waste scavenging. Results reported that the environmental impacts are pervasive worldwide: marine litter, air, soil and water contamination, and the direct interaction of waste pickers with hazardous waste are the most important issues. Many reviews were published in the scientific literature about specific waste streams, in order to quantify its effect on the environment. This narrative literature review assessed global issues due to different waste fractions showing how several sources of pollution are affecting the environment, population health, and sustainable development. The results and case studies presented can be of reference for scholars and stakeholders for quantifying the comprehensive impacts and for planning integrated solid waste collection and treatment systems, for improving sustainability at a global level.

## 1. Introduction

Solid waste (SW) mismanagement is a global issue in terms of environmental contamination, social inclusion, and economic sustainability [1,2], which requires integrated assessments and holistic approaches for its solution [3]. Attention should be paid in developing and transition countries, where the unsustainable management of SW is common [4]. Differences should be highlighted between developing big cities and rural areas, where management issues are different, specifically regarding the amount of waste generated and the SW management (SWM) facilities available [5]. However, both suffer negative economic legislatives, political, technical and operational limitations [6].

Uncontrolled disposal generates serious heavy metals pollution occurring in the water, soil, and plants [7], open burning is cause of CO, CO_2_, SO, NO, PM_10_ and other pollutant emissions that affect the atmosphere [8], waste picking within open dump sites pose to serious health risk people working on these areas [9], release of SW in water bodies improve the marine litter globally, enhancing environmental contamination [10]. Therefore, SW mismanagement is cause of sever and various environmental and social impacts, which do not allow improvements in sustainable development.

Achieving both economic growth and sustainable development involves reduction plans of the global ecological footprint, changing the way of produce-consume-waste of goods and resources [11]. The material footprint of developing countries grew from 5 t inh^−1^ in 2000 to 9 t inh^−1^ in 2017, representing a significant growing in living standards, although its sustainable management is not still included in national regulations [12]. The principles of sustainable development were introduced within the sustainable development goals (SDGs), where 17 objectives were introduced for reducing poverty, improving social equality, decreasing environmental pollution and ameliorating city livability. In particular, the global waste management goals for improving sustainability at global level are: to ensure, by 2020, access for all to adequate, safe and affordable SW collection services; to stop uncontrolled dumping and open burning; to achieve sustainable and environmentally sound management of all wastes, particularly hazardous ones, by 2030 [13].

Many studies reported possible solutions for improving the SWM in developing countries, such as organic waste buyback programs, with compost or biogas production [14], implementation of waste-to-energy plans and technologies [15], waste-to-energy in parallel with recycling of glass, metals, and other inert [16], production of energy from biomass waste by making briquettes [17], involvement of the integration of waste pickers with legal incentives [18], among others. However, many barriers still remain for improving formal collection, treatment and final disposal [19]. Therefore, environmental contamination remains a big issue worldwide, while common solutions should be identified and implemented considering SWM patterns appropriate for each context.

Many reviews were published about SWM in developed and developing countries and about environmental contamination from waste. In particular, about char fuel production [20], management of waste electric and electronic equipment (WEEE) [21], food waste management [22] and treatment [23], recycling of used batteries [24], inclusion of the informal sector [25] and the risks that such activity pose for vulnerable informal workers [26], atmospheric pollution due to SWM [27], household hazardous waste management [28] and healthcare waste (HW) management [29], among others. The novelty of the narrative review presented in this article is its focus on the integrated assessment of these waste streams, analyzing the global issues affecting the environment and the public health, giving attention to the operational risk of the informal recycling sector. Concentration of contamination in water, air and soil are provided, as well as waste quantities and amounts dumped in developing cities or recycled by the informal sector. Results allow suggesting directions for future SWM improvements, considering its planning as an integrated system and providing examples of the consequences of its inadequate implementation.

The paper is divided in three main sections: the first analyzes the environmental impacts due to unsustainable management of municipal SW (MSW), WEEE and used batteries, waste tires, C&D waste and other hazardous and industrial wastes; the second is focused on the informal recycling, analyzing main risks due to waste picking and opportunities for its inclusion within the formal SWM system. The last section is a critical discussion of current and future challenges for improving environmental quality at global level, identifying the opportunities due to SWM selective collection and treatment systems. Finally, some suggestions are provided, according to the literature review.

## 2. Methods

**Figure 1:**
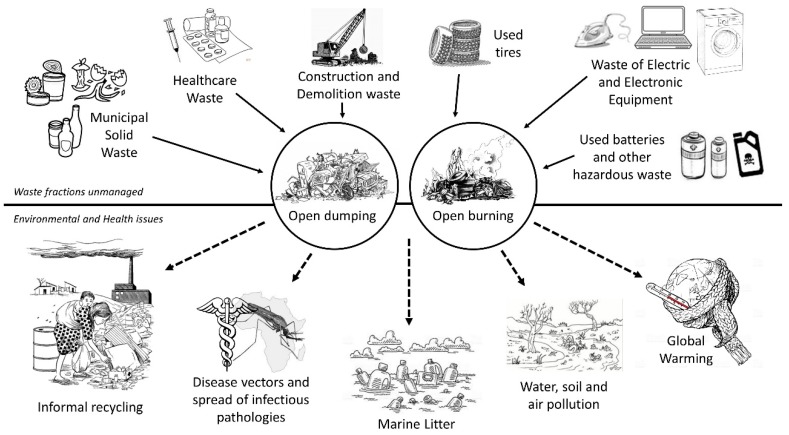
Theoretical framework of the review: source of contamination due to SW mismanagement.

This article reviews the open dumping and open burning of waste, main practices implemented for waste treatment and disposal in developing countries, involving many environmental and health impacts [30,31,32]. Such unsustainable practices include every waste fraction, such as MSW, HW, construction & demolition (C&D) waste, used tires, WEEE, used batteries, and industrial waste, each one spreading specific contaminant concentrations in soil, water and air environments. Waste pickers work within these sites for collecting recyclable materials that are sold in local markets. Though this informal practice allows decreasing the amounts of waste inflow into water bodies and open dumps [33,34], it is also a hazardous activity that improves health and occupational risks [35,36]. Therefore, concerning waste open burning and open dumping, the narrative review presented in this article explores environmental impacts due to unsustainable SWM, such as water, air and soil pollution, health and operation risks, global warming potential (GWP) and marine pollution. The theoretical framework of the review is schematically reported in Figure 1.

The scientific literature considered was collected from three main databases: Scopus, Web of Science and Science Direct. The keywords used for reviewing the literature were the ones that refer to the issues concerning solid waste management in developing countries, therefore combining the keywords “solid waste” and “developing countries” with: open burning, open dumping, informal recycling, health risk, environmental contamination, air-water-soil pollution, C&D, HW, WEEE, used batteries, industrial waste, marine litter. Only papers wrote in English were considered. The scientific articles were reviewed during the months of January and February 2019, analyzing only the literature from 2002 to 2019. Case studies and reviews were considered for the research, with particular focus on developing cities and contaminated area in Latin America and the Caribbean, Africa, Eastern Europe, Middle East, Asia and Oceania. Developed countries were considered only for specific case studies, such as fire of waste tires in final disposal sites and the comparison of same issues detectable worldwide, such as the marine litter in the Mediterranean Sea. Treatment technologies and collection systems were not assessed in terms of contribution of pollution and health risks.

## 3. Environmental and Social Issues due to SW Mismanagement

### 3.1. MSW open Dumping

In developing countries, the management of SW is worsened by unsustainable practices that improve the environmental contamination and the spread of diseases. In particular, the open dumping in uncontrolled sites, open burning of waste fractions and the mismanagement of the leachate produced in final disposal sites, are the main issues detectable [37]. The situation is worsened in slum areas with additional problems of high-density population, traffic, air and water pollution. Uncontrolled disposal in open spaces near water bodies are issues widespread in these contexts, which corresponds to public health issues [38]. Concerning open air final disposal, the main environmental impacts detectable are:

- visual impacts,
- air contamination, odors and green-house gasses (GHG) emission,
- vectors of diseases,
- surface water and groundwater pollution.

These issues are visible worldwide. In Banjul (Gambia) the dump site is located in a densely populated area, visible to the residents [39]. It has a negative visible impact on inhabitants and tourists visiting the country. In particular, the smoke from burning debris is the biggest issue, which covers parts of the residential areas, affecting also the life quality of the population. Indeed, the citizens are affected by the smoke from burning debris and the smell of decomposing waste. The nuisances are worst during the rainy period as the area becomes infested with flies and insects. Run off from the dump site with contaminants dissolved inflow into water bodies, while the leachate contaminates the soil and groundwater. Moreover, environmental contamination is due to the high level of fecal and total coliform that polluted the wells located near the site. The households that live around the dump site use well water for various purposes, although with high level of coliforms attributed to the proximity to the dump site [39].

In Cambodia, in the capital city Phnom Penh, where the MSW management (MSWM) system lacks regulation, households commonly burned, buried, or dumped about 361,000 tons of MSW in 2008, and 635,000 tons in 2015 [40]. In Thailand, more than 60% of the SW final disposal was carried out by open dumping. In 2004 there were 425 disposal sites, of which 330 open dumps, the majority of disposal sites received around 25 tons of waste per day, while only the landfills of Bangkok received about 4500 tons per day [41]. In the West Bank Palestinian territory, in 2005 was estimated that the MSW generated was about 2728 t per day, while in 2001 there were 133 MSW dumpsites, open burning activities at 116 sites and burial at 13 sites; 64.9% of the population was aware of the environmental issues and impacts associated with open dumpsites, and 41.6% thought that they were suffering from the final disposal sites [42]. In Abuja, the capital city of Nigeria, more than 250,000 tons of waste were generated per year in 2010. There were four major disposal sites under its management, closed in 2005 due to odors, air pollution and burning wastes at the site. Moreover, percolation of leachate from the buried waste flowed to the surface, especially during rainy seasons [43]. In Maputo, administrative center of Mozambique, with about 1,200,000 inhabitants and where about 0.5 kg of waste per inhabitants are generate daily, the MSW is transported to the official dumpsite of the city, in operation since more than 40 years. The area is of about 17 ha, with heights that achieved 15 m; open fires and auto ignition of the waste are common issues, exacerbated by the more than 500 waste pickers collecting recyclables waste at the dumpsite [44]. Therefore, SWM issues are common worldwide, with environmental burdens and hazard for the population.

The landfill leachate generates in open dump sites contains concentration of organic carbons, ammonia, chloride, heavy metals [45], as well as high concentrations of fluoride, chloride, ammonium–nitrogen, biological oxygen demand (BOD) and chemical oxygen demand (COD) [46]. For instance, the MSW dumped at Mathkal dump site (Kolkata, India), is affecting the degradation of water quality in and around dumpsite area: Cd and Ni are detectable in leachate, improving groundwater contamination; the metals Pb, Cd, Cr and Ni are characterized as toxic for drinking water, and the concentration of these components increases near an unsanitary landfill and may lead to serious toxic risks. Indeed, It has been reported that the concentration of Cr, Cd, and Mn were higher in the groundwater due to leachate, affecting the life of the population and the quality of the environment [47].

In Chennai city, the capital of Tamil Nadu, India, where more than 3200 t d^−1^ of SW are generated, the leaching of heavy metals in the water imposes serious health risks to humans. Heavy metal concentration of the soil samples at various depths ranges from 3.78 mg kg^−1^ to 0.59 mg kg^−1^ at a depth of 2.5 to 5.5 m, with concentration higher in the top soil up to a depth of 5.5 m (sandy clay layer). Therefore, the concentrations of heavy metals decreased with increasing soil depth, demonstrating the influence of the dumping activities [48]. In Nonthaburi dumpsite, Thailand, the concentration of heavy metals was detected in boreholes and runoff. Within the runoff and the groundwater, the concentrations of chrome, cadmium, lead, nickel and mercury, are always 10 times above the limits introduced by the World Health Organization (WHO) for drinking water [49]. In Tiruchirappalli district, India, the MSW generation is about 400–600 tons per day and it is served by an open dumping site located 12 km from the city. The leachate shows that the range of COD range to 29,880–45,120 mg L^−1^ and the BOD_5_ / COD ratio was less than 0.1. Based on the average concentration, the quantity of lead and cadmium were 5 and 11 times higher the soil contamination limits. The presence of heavy metals (Pb, Cu, Mn, and Cd) in soil sample, undetectable in the near areas, indicates that there was appreciable contamination of the soil by leachate migration [50].

In Table 1 pollutant concentrations in soil, runoff and groundwater in eight different case studies, compared with the limits imposed by international organizations for soil and water quality are reported. In the case studies reviewed, the analysis was implemented at a distance variable from 20 to 400 from the final disposal sites. Data about runoff and groundwater contamination were compared with drinking water limits since, in low-middle income areas, groundwater is the most use for drinking without adequate treatments. Results reported always a correlation between leachate and environmental contamination. Heavy metals are always the ones persistent within the samples, also 10 times more than the limits suggested by the WHO, with high concentrations of COD. So, open dumping poses surrounding population to serious health risks.

Another environmental issue due to organic waste open dump is the GWP due to waste anaerobic degradation. Methane gas is a by-product of landfilling MSW; since MSW is mainly disposed of in open dump sites, the generated methane is released directly to the atmosphere. Experimental studies indicate that the anaerobic biodegradation of MSW organic waste generates about 200 Nm^3^ of methane per dry tons of biomass [58]. Methane is one of the most important gas that improve the GWP, 25 times higher than CO_2_ [59]. Therefore, open dumps and uncontrolled landfills are direct source of GHG.

As a comparison, GHG emissions from waste landfilling were estimated per type of final disposal site: open dump, conventional landfills with energy recovery, and landfills receiving low-organic-carbon waste. The results showed that about 1000 kg CO_2_-eq. t^−1^ are generated from an open dump, 300 kg CO_2_-eq. t^−1^ from a conventional landfilling of mixed waste and 70 kg CO_2_-eq. t^−1^ for low-organic-carbon waste landfills. If compared with the emissions due to provision of energy and materials to the landfill, estimated to 16 kg CO_2_-eq. t^−1^, it can be stated that open dump cause a GWP at least 50 times higher than the total MSWM system [60]. In Beijing City, where more than 60% of the waste is disposed of in sanitary landfills, an environmental impacts assessment showed that CH_4_ emission is the most dominant contributor to GWP, with the annual amount of 55,000 tons; the landfills contribute the most to the impact potentials mainly due to methane emissions [61]. In India, most of the SW are disposed of by landfilling in open dump sites, generating large quantities of CH_4_. At national level, It was estimated that the methane emission from MSW disposal varies from 263,020 t in year 1980 to 502,460 t in year 1999 [62], increasing rapidly during the years.

Therefore, the mitigation of pollution and GHG emission can be obtained through the recovery and conversion of organic component to energy or compost. The main role is played by policy interventions, which should act through the incorporation of the waste management hierarchy considering direct and indirect impacts that would reduce the global carbon footprint [63].

### 3.2. Marine Litter

Open dumping cause surface water pollution due to leachate mismanagement and material uncontrolled flows. A visible impact that is affecting the seas and the oceans globally is the marine littering, which is mainly caused by plastic waste [64,65]. Marine litter is defined as manufactured or SW entering the marine environment irrespective of the source. The range and scale of impacts from marine litter are diverse [66]:

- Environmental (ingestion, poisoning, blockage of filter, physical damage of reefs and mangroves, among others),
- Social (loss of visual amenity, loss of indigenous values, risks to health and safety),
- Economic (cost to tourism, cost to vessel operators, losses to fishery, costs for cleanup, animal rescue operations, recovery and disposal),
- Public safety (navigational hazards, hazards to swimmers and divers, cuts, abrasion and stick injuries, leaching of poisonous chemicals, explosive risk).

**Figure 2:**
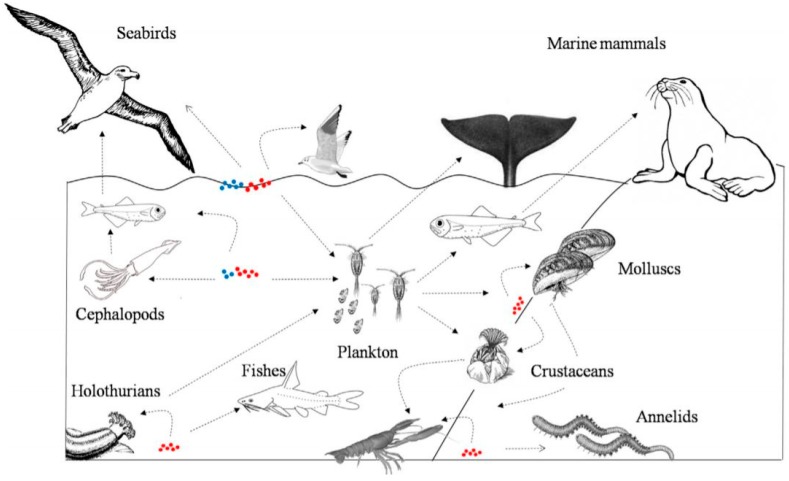
Schematic analysis of the trophic chain of micro-plastic in the marine environment, for explaining plastic direct and indirect ingestion [69].

About 80% of marine litter generation is mainly caused by the mainland, by the rivers that inflow into the seas [67]. Therefore, open dumping can be considered as the first cause of pollution of the oceans. More hazardous is the generation of micro-plastics: Once in the ocean, most plastics tend to stay at or close to the surface where the photo-chemical, mechanical and biological processes degrade larger items into smaller, less than 5 mm, forming microplastics [68]. Potentially, microplastics are ingested when present in the marine environment and tend to float on the sea surface. There, they can be ingested passively or actively by a wide range of organisms [69]. A simple scheme has been provided by do Sul et al. [69], where the definition of direct or indirect ingestion of micro-plastic, which can affect human health, is clarified (Figure 2).

A study published in 2019 reported that, in the Mediterranean Sea, microplastics are 94.6% in number and 55%_wt_ of all plastics whereas meso-plastics represented 5.3% in abundance and 45% in weight of all plastics. In this study, only 1 macro-plastic was sampled, which represented 0.1% in abundance of all plastics and weighed five times more than all the collected plastics together [70]. It means that the amounts of micro-plastic are increasing, improving the risk of direct and indirect intake within the trophic chain, achieving human feeding. Moreover, a study conducted in the Pacific Ocean, discovered plastics from the 1960s, which means that the marine littering and the pollution of the sea is 60 years old, improving the amount of microplastics detectable into the marine environment [71].

The implementation of sound waste management collection and disposal practices, involvement of manufacturers, and behavior change are key aspects of any solution. At an intermediate stage, innovation is needed around the litter generation points: upstream, redesigning goods for reducing generation quantities; and downstream, improving collection and treatment systems. Long-term technical solutions for recovering the existing used plastics in the world’s seas should also be implemented [37]. Finally, a specific focus on low-middle income countries should be considered, since they are the main source of pollution although the generation rates are the lowest.

### 3.3. MSW open Burning

Waste open dumping is not the only environmental burden due to waste mismanagement. The combustion of waste with any precaution generates also contaminants, improving health risks to the population [72]. Polychlorinated dibenzo-*p*-dioxins (PCDDs), polychlorinated dibenzofurans (PCDFs), and polychlorinated biphenyls (PCBs) were detected in soils around dumping sites in The Philippines, India, Cambodia, and Vietnam [73]. Uncontrolled combustion, generation of methane gas, and low-temperature burning are major factors for the formation of dioxins in dumping sites. Considerable loading rates of PCDD/Fs in the dumping sites of these countries (200–4000 tons per day) were observed, ranging from 0.12–35 mg _TEQ_ yr^−1^ [73].

Open dumping sites in Surabaya and Palembang, Indonesia, have concentrations of PCDD/Fs and dioxin-like polychlorinated biphenyls (DL-PCBs) in soil of about 61,000–310,000 fg _TEQ_ g^−1^ (dry weight) and 6300–32,000 fg _TEQ_ g^−1^, respectively. Low levels of PCDD/Fs and DL-PCBs, ranging from 75 to 98 and 0.32 fg _TEQ_ g^−1^, respectively, were observed in soil for an open dumping site that included a top cover layer of soil. The difference in concentrations can be explained by the fact that open burning of waste is the source of PCDD/Fs and DL-PCBs. A sensitivity analysis implemented in this area found that the maximum emission factor could be 5,600,000 fg _TEQ_ g^−1^ [74].

A controlled incineration that treated about 100,000 t of MSW per year, required for a city of about 350,000 inhabitants who generate about 0.8 kg MSW per day, generates about 40,000 fg _TEQ_ m^−3^ [75], which is equal to 24 mg _TEQ_ yr^−1^, considering a production of 6000 m^3^ of combustion gases per ton of waste burned. Therefore, open dumping can generate more quantities of dioxins per year than an incinerator, also with uncontrolled leachates, diseases vectors, odors and GHG, affecting the environment and population’s health.

In the Municipality of Huejutla, Mexico, approximately 24% of the total waste generated was burned by households, of which 90% in rural areas, where there was not an MSW collection system. This practice generates environmental contamination and contributes to the GWP by the production of black carbon (BC). It has estimated that about 8,882 tons of waste are burned per year, producing 1.97 kg BC t^−1^, 11.9 kg PM_10_ t^−1^, and 9.8 PM_2.5_ t^−1^ that contributed for 17.5 t BC y^−1^ (38,553 t CO_2_-eq per year), 105.7 t PM_10_ y^−1^ and 87.0 t PM_2.5_ y^−1^, for a total of 313.7 kg CO_2_-eq y^−1^ per capita. The results showed that the CO_2_-eq from BC emitted by waste open burning was more than 15 times larger compared to CH_4_ potentially released from the decomposition of equivalent amounts of combustible organic waste deposited at the dumpsite [76]. In another study, it was found that the majority of PM generated by waste open burning had smaller sizes (PM_1_) compared to PM_2.5_ and to PM_10_. In particular, the PM size were 0.35 µm, with about 63.0 µg m^−3^ generated, and 0.45 µm, with 67.8–87.7 µg m^−3^. Therefore, 0.45 µm had the highest peak concentration among all the compounds. The study demonstrated that the smallest-sized particles (0.35 and 0.45 µm), which represents the most hazardous for the population health, constituted the greatest percentage of total PM emissions, founding that the concentration of ultrafine particles represent another source of hazard for population health [77]. The review of the scientific literature indicated that open burning should be avoided and replaced with appropriate and sustainable technologies for reducing environmental pollution and public concerns. Know-how is required, as well as financial support for improving waste recovery and final disposal at global level.

### 3.4. Health and Environmental Risks due to HW Mismanagement

SW is not only municipal. There are various fractions hazardous for the environment and the population health that are generally mismanaged in developing countries. One of these fractions are the HW [78]. The term HW includes all the waste generated within health-care facilities. In addition, it includes the same types of waste originating from minor and scattered sources, including waste produced during health care undertaken at home. Between 75% and 90% of HW is comparable to MSW, so “non-hazardous” or “general HW”. The remaining 10–25% of HW is hazardous and may pose a variety of environmental and health risks [79]. Details about HW fractions is reported in Table 2.

Open dumping is the most common method of HW disposal in developing countries [80,81,82], although several authors suggest sterilizing the HW at the point of generation in order to eliminate infectious substance and improve safety management [83]. Open dumping is the lowest cost option for low income countries, although it is an uncontrolled and inadequate disposal, since the waste can be accessible to waste pickers and animals and the generation of pollutant is not monitored. In this way, HW transmits infectious pathogenic micro-organisms to the environment either via direct contact, through inhalation, ingestion, or indirect contact through the food chain. Burning is aimed to reduce the volume of waste and its infectious effect, however, uncontrolled burning activates are potential source of toxic emissions like PCDD/F, among other pollutants [84].

**Figure 3:**
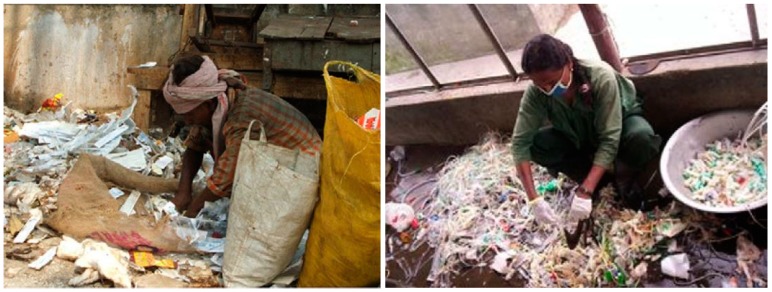
Informal HW scavenging in Dhaka, Bangladesh [87].

In the West Bank (Palestine), a study shows that 82.2% of HW is disposed of in (unsanitary) dump sites and only 17.9% of healthcare facilities dispose of their waste more than 7 times a week, the frequency recommended by the WHO. Therefore, the final disposal locations in the West Bank are uncontrolled final disposal sites, which are randomly distributed throughout the region, with poor precautions for transporting and colleting the HW [85]. In Ibadan, Nigeria, more than 60% of HW handlers did not discriminate between HW and MSW during collection and handling stages. Similarly, 66% dispose of HW with MSW at the final disposal site (open dumps). Incidences of contacting diseases are prevalent among waste handlers, compared to incidence of other hospital staff, with high incidences of viral blood infections, such hepatitis B and C. Within the open dump sites, technical and hygienic considerations are neglected or absent. For instance, several waste pickers were observed collecting HW for reselling materials considered recyclable, to pass-on to unsuspecting low-income patients. Moreover, leachate from waste disposal sites could be infiltrating and contaminating groundwater resources [86]. In Dhaka, Bangladesh, HW is collected by waste pickers who sort the waste through the bins searching for recyclables and reusable items (syringes, blades, knives, saline bags, plastic materials and metals). Scavenging activities were again observed sorting through the open dumping disposal sites, increasing the risk of diseases (Figure 3). The study reported that both scavengers and recycling operators had any knowledge of the risks from HW exposure. Employers of recycling operators did not consider occupational health and safety training for their employees. The situation was still more worrying among the marginal groups of the society [87].

The lack of appropriate HW management systems and disposal facilities in Dhaka is largely due to inadequate economic resources and legislation. This leads to the persistence of inappropriate practices such as the discharge of chemical waste into the general sewerage system or dumping into near land. HW was found to have been dumped in MSW bins, and finally disposed of on general landfill sites, which may contaminate the ground water and improve operational risks. It was observed that, during the rainy season, leachate from dumps used for HW infiltrated into water that was being used for washing and for household purposes, as well as for agriculture [88].

Therefore, in low-income countries, HW management is an environmental and social issue that spread the risk of disease and pollution. Disposal strategies involve sorting HW at the healthcare facilities, and then transporting the infectious HW to safe disposal sites, where it is treated by incineration or other technologies and the residual product landfilled. Every treatment technology has drawbacks, with incineration creating atmospheric emissions, and other treatments not able to handle all types of waste. The best way to control the impact of HW is to train healthcare workers along with the implementation of standardized HW streams and disposal bin colors, which can ensure a selective collection of the waste, improving the efficiency of treatment and final disposal [89]. Good results were obtained in San Salvador (El Salvador), where an information campaign was implemented for the employee of tertiary hospitals. Before the activity, the employees disposed of common waste in containers for infectious waste, increasing the hospital’s financial and operational burden, while after the project the quantities were halved, demonstrating the good compliance of the operators and of the activities implemented [90].

### 3.5. Open Dumping and Burning of WEEE and Used Batteries

Global WEEE generation has reached approximately 41 million tons in 2014, increasing at a rate of 3–5% every year [91]. The production of WEEE was correlated with the GDP, while there is no significant correlation or trend with the population. If this waste is properly recycled, it could offer an opportunity for the recovery of copper, gold, silver, palladium and other metals with an estimated value of USD 48 billion. In particular, the concertation of metals in the WEEE is significantly higher than in the natural ores that these metals are mined from (for Au it is almost 130 times higher) [91]. WEEE are classified into six different types of waste [92]:

- Temperature exchange equipment: refrigerators, freezers, air-conditioner, heat pump,
- Screens and monitors: televisions, monitors, laptops, notebooks, tablets,
- Lamps: fluorescent lamps, high-intensity discharge lamps,
- Large equipment: washing machines, clothes dryers, electric stoves, large printing machines, copying machines, photovoltaic panels,
- Small equipment: vacuum cleaners, toasters, microwaves, ventilation equipment, calculators, radio, camera, toys, medical devices, small monitoring and control equipment,
- Small telecommunication equipment: mobile phones, GPS, telephones.

**Figure 4:**
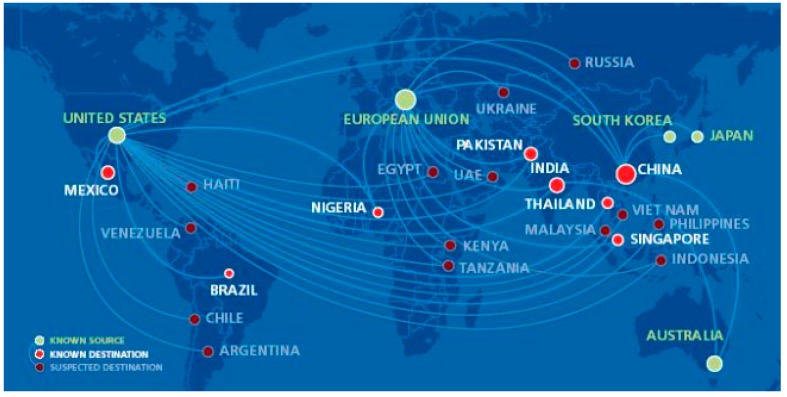
Estimation of the legal and illegal WEEE flow from high-income to low-income countries at global level [94].

Developing countries are producing WEEE double than developed countries. It is also estimated that the developing and developed countries will discard 400–700 million obsolete computers by 2030. Moreover, developed countries are also exporting their WEEE to developing countries for dumping, causing serious environmental and social concerns. The majority of WEEE are being sent to Africa or Asia [93]; in Figure 4 are reported the estimated flows of the waste from high income to low income countries.

WEEE is becoming a source of income for the industries and creates new jobs. However, in developing countries WEEE are mainly disposed of in open dump sites, burned without properly precautions and managed by illegal actors [95,96]. In India, Bangalore city generates 18,000 tons of WEEE per year, thousands of which are landed illegally every year [93]. In Lagos State, Nigeria, near an open dump site where WEEE and used batteries are disposed of with MSW, the heavy metal concentrations in well water and soil were investigated during the dry season [97]. Results reported that concentrations in wells were Pb 2.77 mg L^−1^, Cd 0.035 mg L^−1^, Zn 0.948 mg L^−1^, Cr 0.520 mg L^−1^ and Ni 1.45 mg L^−1^, while Ni concentrations in soils ranged from 35.45 mg kg^−1^ at a depth of 15–30 cm in the wet season to 85.43 mg kg^−1^ at a depth of 0–15 cm in the dry season. The elevated level of metals in the well water are correlated with the metal input from leachates resulting from the dumping of WEEE. In fact, significant levels of Pb and Ni were found in well and tap water at the residences, while the concentrations of heavy metals decreased when the sampling distances from the dumpsite increased [97]. Moreover, concentrations of lead, chrome and nickel are generally higher than the ones reviewed in studies conducted near MSW open dumps (Table 1), suggesting that the presence of high amounts of WEEE is cause of heavy metal pollution of water bodies and soils.

In Tijuana (Mexico), a study analyzed the concentrations of Cd, Cr, Cu, Pb and Ni in the soil near an open dump site where end-of-life vehicle (ELV) and WEEE are disposed of, together with the activity of waste pickers who recover the precious metals [98]. The mean concentrations found were 1.4 mg kg^−1^ for Cd, 4.7 mg kg^−1^ for Cr, 304 mg kg^−1^ for Cu, 74 mg kg^−1^ for Pb and 6 mg kg^−1^ for Ni. The results of the geo-accumulation index values show that the site was very polluted with Cu and Pb. The correlation analysis shows a high connection between Pb and Cu, which would be explained if the main source of the polluting heavy metals was the result of electrical wire burning to recover copper. The other two components detected within the study were Cr and Ni, related to the corrosion of junk metal objects and automobile use [98]. Again, in this case study, it is evident that the presence of WEEE is responsible of heavy metal pollution of the soil and therefore of the groundwater used for house uses. Therefore, within Table 1 results of open dumps that also contains WEEE, used batteries and ELV were reported.

Together with WEEE mismanagement, used batteries should be also mentioned. For instance, in Iran, almost 10,000 tons of household batteries were imported, most of them have been discarded in MSW without any separation and sent to sanitary landfills [99]. In addition to environmental and human health risks associated with unsafe disposal of used batteries in MSW stream, their landfilling implies the depletion of valuable resources. It is expected that more than 9000 tons of used batteries have been dumped in municipal landfills of Iran in recent decades. The most concern regarding battery disposal in MSW is directed to the high percentage of mercury, cadmium, lithium, nickel, arsenic and other toxic and heavy metals [99].

The challenges facing the developing countries in WEEE and used batteries management include the absence of infrastructure for appropriate waste management, lack of legislation dealing specifically with these waste fractions, the absence of any framework for end-of-life product take-back or implementation of extended producer responsibility (EPR) [100]. Moreover, the growing rate of WEEE amount in developing countries is destined to increase in the next future [101]: A great amount (almost 50%) of current WEEE yearly generated by developed countries continues to be illegally transferred in developing countries, volumes that remains unknown; New electric and electronic products will substitute soon the current ones, influencing both collected volumes, type of recovered materials and recycling processes; Innovative materials composing WEEE, that are currently not correctly managed during their end-of-life (ending into landfills); some electronic parts in WEEE are not again correctly disassembled or recovered [101]. In summary, many challenging issues of WEEE and used batteries management can be detected in developing countries [102]:

- Quantity of WEEE generated is a major concern due to the lack of infrastructure,
- Inventory assessment of WEEE does not exist,
- Exportation of WEEE from developed countries to developing countries for recycling worsens its management,
- Absence of knowledge regarding the toxic nature of WEEE and used batteries,
- Portion or components of WEEE are often mixed with MSW and disposed of in open dump sites,
- Deficient knowledge of the impacts to human health and the environment,
- Legislation to regulate and control the import and disposal of the generated WEEE do not exist.

Environmentally sound management requires the establishment of collection, transportation, treatment, storage, recovery and disposal of WEEE. Regulatory authorities should have to provide these facilities and for the better performance there should be incentives. Communication campaigns should be oriented to the citizens, in order to improve and incentive the selecting collection of the waste, avoiding open dumping. Furthermore, incentives for municipalities that demonstrate the best results when participating in recycling initiatives should be adopting, in order to motivate the citizens in supporting local management policies and actions [103].

### 3.6. C&D Waste open Dumping

**Figure 5:**
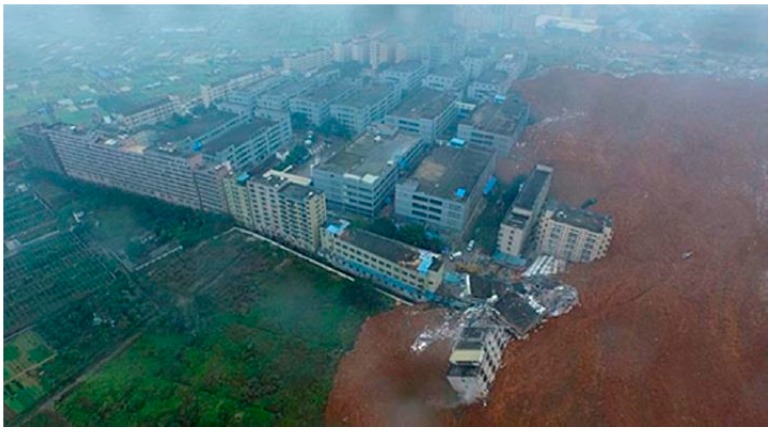
C&D waste landfill landslide in Shenzhen, China [105].

The term “C&D waste” is generally used to refer to the SW generated in the construction sector. More specifically, the term is defined as the waste generated from construction, demolition, excavation, site clearance, roadwork, and building renovation [104]. The main issue due to C&D waste is final disposal site landslides, which can affect the life of the population. For avoiding this impact, the volume of waste dumped in landfills should be reduced, imposing safe operating practices. In particular, 4Rs (reduce, reuse, recycle and recover) policies should be implemented, with hazardous or toxic materials that should be the primary targets [104]. As example, in 2015, a landslide in one of China’s most advanced cities, Shenzhen, killed 73 people and damaged 33 buildings, in the absence of heavy rainfall or earthquakes (Figure 5). According to China’s Ministry of Land and Resources, the landslide was triggered by the collapse of an enormous pile of C&D waste [105].

In Thailand, in 2014 the average generation of C&D waste was approximately 4,200,596 tons, which were disposed of in open dump sites. Hazardous and potentially hazardous materials were found, such as:

- asbestos-based materials,
- lead-based materials,
- other materials used for construction (e.g., damp-proofing chemicals, adhesives),
- mercury-containing electrical equipment (e.g., fluorescence lamps, thermostats),
- chlorine fluoride carbides (e.g., air conditioners and refrigerators),
- corrosive, flammable and toxic materials.

Hazardous waste was not separated from non-hazardous waste for proper treatment and disposal. It means that an increasing of the construction sector also contributed to the increasing of environmental pollution [106]. It has been estimated that between 2002 and 2005, an average of 1.1 million tons of C&D waste was generated per year in Thailand [107]. This constitutes about 7.7% of the total amount of waste disposed in both landfills and open dumpsites annually during the same period. Therefore, the generation of C&D waste was affected by a relevant increase. Indeed, recently, the management of C&D waste took attention due to its rapidly increasing and unregulated dumping [108]. Waste generation at a construction site may result from lack of attention being paid to the size of the products used, lack of interest of contractors, lack of knowledge about construction during design activities, and poor materials handling. Generally, 50–80% of C&D waste is reusable or recyclable, so C&D mismanagement represents a loss of valuable economic resources [107].

In Hanoi, Vietnam, processing quantities of informal and formal C&D waste recyclers were revealed [109]. However, current practices lacked appropriate C&D waste classifications and control of waste flows by private companies due to little efficiency or cost saving strategies, low attention for adding value to concrete waste recycling and lack of government legislative and financial support for industry transformation. Illegal dumping occurs in the city boundary, also due to the lack of technology, capacity and economic resources. Many construction sites mix C&D waste such as cement, bricks, steel, and plastics, disallowing the classification and recycling of these fractions [109]. In Malaysia, in the first quarter of 2015, the construction industry contributed 15.1% of the country’s GDP and provided employments to about 10% (1.4 million) of the total workforce in Malaysia. Four key issues were addressed for developing an effective C&D waste management: the increasing amount of waste, environmental impacts, illegal dumping, and lack of national support. In Malaysia, the recycling framework for improving C&D waste management is built following a three-layer approach [110]: At the micro-level, reducing wastes at the source; at meso-level, ensuring that there is a continuous effort in managing wastes, transforming the procurement methods; Finally, at the macro-level, providing monitoring, and coordinating mechanisms to ensure the practice of effective C&D waste management [110].

Therefore, for developing countries with limited financial resources, C&D waste management initiatives and sustainable construction can be achieved through effective utilization of resources, material recovery, and an improved system for waste management. However, the first objective to be achieved is the implementation of strong regulatory initiatives for construction waste management [111]. These practices can reduce the issue of the open dumping, which is worsened by the mix with MSW and informal recycling that operates in these uncontrolled areas.

### 3.7. Diseases Exposure due to Used Tires open Dumping and Burning

Tires that are used, rejected or unwanted are classified as ‘waste tires’, as well as tires intended to be used for retreading or recycling. This type of waste is composed of steel, rubber and textiles, and the volume depend on the use of the tires. Three main issues should be addressed concerning waste tires:

- big volumes, which reduce the useful life of the sanitary landfill and improve the transportation costs,
- open air burning of these materials, which contaminate the environment improving population health risks,
- presence of disease vectors, such as insects or rodents, which live inside the holes and furrows of the tires.

In developing countries, limited data reliability on used tires availability and collection is common, as well as small activities of uncontrolled waste recovery, with cases of illegal dumping [112]. One of the most hazardous problems regards the spread of Dengue, which is currently one of the most important diseases in tropical areas. About 2.5 billion people live in areas of risk and many millions of cases occurring each year [113]. A study assessed the breeding mosquito larvae, identifying the dengue vectors distributed in Tamilnadu (India). Totally 118 water containers were inspected, among which 38 containers were recorded as positive for dengue vector. Among all type of containers analyzed, cement cistern, mud pot and used tires were positive for the mosquito larvae [113]. Therefore, the final disposal in open dump sites of waste tires should be avoided for reducing the spread of Dengue diseases in topical areas.

Another impact that affect the population health is the uncontrolled burning of waste tires. In Nepal, where the uncontrolled open-air burning of waste tires is practiced also during political agitation, a study was conducted to provide background information for assessing the environmental pollution due to tire fires [114]. The effect of the tire fires on air is a major concern, because they release potentially hazardous gases such as CO, SO_2_ and NO_2_ as well as polyaromatic hydrocarbon (PAH) and volatile organic compounds (VOC). CO is formed whenever carbon or substances containing carbon are burned with an insufficient air supply. Tire fires, apart from intense heat, give off BC with CO emission. Results of the research reported that the emission levels of CO from different type of tires were 21–49 g kg^−1^, SO_2_ emission was found to be 102–820 g kg^−1^, while NO_2_ emission was 3–9 g kg^−1^ [114]. These emissions can be compared with wood combustion, in order to have an indication about the pollutants of major concerns due to tires burning. Emissions of pollutants from residential wood combustion sources in wood-burning stoves are NO_x_—NO_2_ 0.5 g kg^−1^, SO_x_—SO_2_ 0.2 g kg^−1^, CO 83–370 g kg^−1^ and PM 0.6–8.1 g kg^−1^ while in fireplaces are of NO_x_—NO_2_ 1.8 g kg^−1^, SO_x_—SO_2_ absent and CO 11–40 g kg^−1^ [115]. Therefore, it is evident that the generation of sulfur compounds generate more environmental concern in terms of quantity produced if compared with wood fire.

Open fire issues are also detectable in high-income countries, where waste tires landfills are still an issue. A large and uncontrolled fire of a tire landfill started in Toledo (Spain), and experimental analysis were implemented for measuring the potential impact at local and regional levels [116]. Outdoor and indoor measurements of different parameters were carried out at a near school, approximately 700 m downwind the burning tires. Among metals, ZnO and Co were 21 and 92 times higher than an area far from the open fire, reaching 933 µg m^2^, compared with 13 µg m^2^ in the farther zone. Increases of SO_2_ and PM_10_ levels were also detected, with sulfate concentrations of 1371 µg m^2^, 11 times higher than the control zone [116]. A similar study was conducted in the Iowa city landfill, (United States), where the outdoor concentrations of pollutants generated from 18 day tire fire were assessed [117]. The study estimated maximum concentrations of tire fire PM_2.5_ smoke at distances of 1, 5 and 10 km of 243, 55 and 26 mg m^−3^, respectively. SO_2_, PM_2.5_, BC, and air toxic VOC had also high concentrations if compared with areas far from the fire. In another study, where tire smoke was investigated, BC, biphenyl, benzene, benzaldehyde, PM, and CO were highly ranked hazards [117].

These environmental issues due tire open dumping and open burning should be addressed in an integrated manner, in order to avoid these practices. One suggestion provided by various authors is to introduce the EPR, to ensure environmentally effective management of end-of-life waste, following 4Rs [118]. This regulation tool wants to prevent waste formation and promote source reduction. If this is not possible, waste should be reused, recycled, and then recovered for energy, while landfilling should be avoided. Accordingly, the tire EPR system should reduce the generation of tire waste, facilitate its reuse, promote recycling and other forms of material recovery and, finally, incentive the energy recovery, although LCA studies confirmed that the material recycling of tire waste provides greater environmental benefits than energy recovery [118,119,120].

### 3.8. Industrial Waste open Dumping

Finally, environmental contamination due to industrial waste mismanagement should be considered, since they are mostly hazardous. There are many different types of hazardous industrial waste, as well as source of contamination, such as mine tailings, fly ash, waste from the production of chemicals (e.g., phosphoric acid), residues from coal mining, acidic waste rock, carbide slag, among others [121].

In a tanneries area located in Ranipet (India), where chromate chemicals were manufactured, a large quantity of hazardous SW was stacked in open dump sites. This practice resulted in fast migration of the contamination to the groundwater, with levels of chromium up to 275 mg L^−1^, 1000 times higher than the recommendations of the WHO for drinking water. The findings are of relevance for addressing the groundwater pollution due to indiscriminate disposal practices of hazardous waste [122]. A primary lead smelter operated in Santo Amaro City in Brazil, from 1960 to 1993, leaving approximately 500,000 tons of industrial waste containing 2–3% of lead and other toxic elements that contaminated the soil. The waste was deposited on the grounds belonging to the smelter, without any cover or precaution. In 2008 the average concentrations in soil were 1040 mg kg^−1^ for Pb, 2.73 mg kg^−1^ for Cd, 22 mg kg^−1^ for Ni, 295 mg kg^−1^ for Zn and 5.2 mg kg^−1^ for As, with a strong correlation among Cd, As and Zn. Therefore, the contamination due to heavy metals persists during 15 years, affecting the population surrounding the site, in particular the youngest [123]. In Dar es Salaam City (Tanzania), industrial waste (paints, pharmaceuticals, rubber, plastic, metal scraps, packaging materials, among others) are disposed with MSW within open dump sites. The dump of hazardous and non-hazardous wastes poses serious public health and environmental issues, since rainwater leach from the waste to the groundwater contaminating the surrounding areas [124]. Same issues regard the agriculture industries, with the production of waste related to pesticide containers and spry solutions. For example, in rural areas of Greece, farmers are used to dumping the empty containers on irrigation canals or in the field, sometimes burning or troughing them in others waste open dumps, generating river, soil and atmospheric contamination [125].

These results show that also industrial waste management is an underestimated issue and should be treated with appropriate methodologies and technologies. SW collection represent the first step, avoiding open dumping, after which a selective collection should be implemented in order to allow the recovery of valuable materials. Afterwards, incineration, chemical physical treatments, and appropriate final disposal should be implemented in function of the waste fraction generated, such as waste oils and solvents, batteries, emulsions and chemicals, sludges and refractory materials, among others [126].

## 4. Informal Recycling and Social Inclusion

Worldwide, there is a considerable presence of the informal sector in SWM, particularly in low-middle income cities where formal selective collection systems for recyclable materials are not still developed [127]. Informal activities tend to intensify in times of economic crises and where imported raw materials are quite expensive. However, its inclusion in formal SWM systems remain a problematic issue and considerable attention from NGOs and scholars is arising for solving such problem [37].

**Figure 6:**
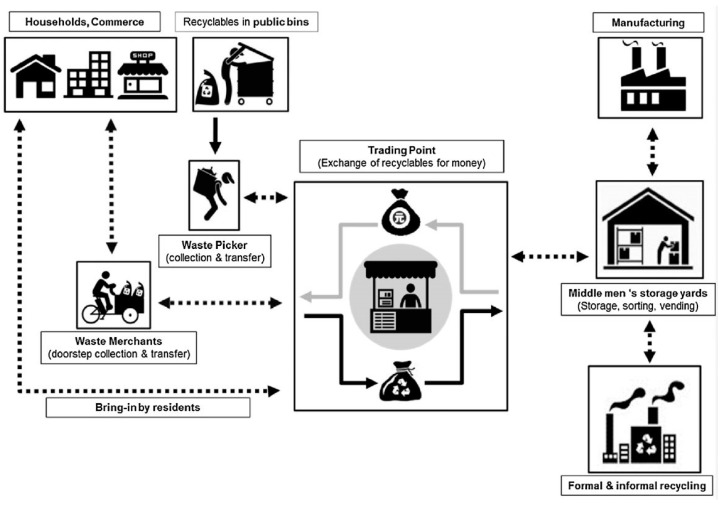
Informal recycling chain in China, as schematically depicted by [128].

In Figure 6 is reported a simplified scheme that represent the selective collection chain of the informal sector [128]. The structure is of a specific case study in China, however, the structure is similar worldwide. The informal pickers collect the waste in open dump sites, bins, roads and households for segregating recyclable materials. These people can be organized or alone, with or without transportation means, and can be merchants or simply pickers. The waste is then sold to trading points that collect the waste and sell it again to formal or informal recyclers or directly to manufactures. This structure can be recognized in many case studies within the scientific literature.

Many studies were implemented and published, in order to assess how the informal sector could be included in the formal management or recognized by the local population. For instance, in Ulaanbaatar (Mongolia), the informal sector operates in t informal neighborhoods. In these areas, illegal dumping is common, and some open fields became uncontrolled disposal sites, with waste pickers working and living near these areas. In 2004, the World Bank estimated that about 5000 to 7000 informal recyclers worked in Ulaanbaatar, and today this number could be higher due to the increase in city’s population. A study in the city revealed that most waste pickers have also higher education at a university, suggesting that the activity is due to many factors (e.g., lack of work). Informal waste pickers select recyclable materials and bring them by foot to secondary dealers for obtaining an income, who then sell larger quantities to the respective recycling industries [129].

In Blantyre (Malawi), MSW is disposed of in pits, along the road-side, or in the river. Waste pickers process and transform recyclable materials reducing the amount of waste disposed at dumpsites and decreasing the use of virgin materials needed for manufacturing. However, waste pickers are rarely recognized for their contribution. The two waste categories selected by the pickers are plastic and metals. No data are available for quantifying the number of waste pickers, however it was estimated that the maximum quantity of waste selected per day was about 20–30 kg d^−1^ [130]. In Harare, Zimbabwe, where the quantities of waste generated within the city are not known, the informal sector operates, mainly in open dump sites. Indeed, the waste collected by the formal collection is disposed of in dumpsites, where about 220 waste pickers worked. Waste pickers required a license to enter the dumpsite and had to wait for a worker’s signal before they could start recovering materials. It was estimated that the informal recycling sector recovered about 6–10% of waste deposited at the final disposal site (about 27–50 tons per day). Competition with others pickers was considered as the major challenge for the collectors, as well as workplace health and safety and discrimination among the population [131]. In Zavidovici (Bosnia Herzegovina), where solid waste is disposed of in open dumps, informal recycling represents the main income-generating activity for a group of ethnically discriminated households. These families contribute to the recovery of iron, copper, plastics and cardboard from MSW, reducing the waste inflow into the dump sites [132]. Finally, in Iloilo City (The Philippines), where some 170 tons of waste (about 50% of the total generated) are disposed of in an open dumpsite, approximately 300 households recover recyclable materials for selling them in local markets. A pilot project with international NGOs was implemented, in order to convert the organic waste into energy through briquette production. Results of the study show that the integration of the informal sector in the production of biomass briquettes can be a good option for implementing integrated plans for including informal recyclers, especially in areas where their activity is forbidden, as in The Philippines [133].

In Table 3, seven case studies are compared in order to assess which are the number of pickers, their organization, its source of waste, the quantities and the fractions collected per day and the main issues detected by the studies. Results reported that waste pickers operate both in low income (Zimbabwe) and high-income countries (China). Mostly, informal sector collect waste from uncontrolled open dump sites and are not recognized or organized by the local municipalities. Waste pickers can collect from 14 to 60 kg of recyclable waste per day, which comprehend WEEE, MSW and HW.

Regarding the environment and the recovery of resources, the benefits are evident in many cities. In some places informal-sector service providers are responsible for a significant percentage of waste collection. In Cairo (Egypt), the informal recycling is implemented since the recyclable waste recovered are sold to the private companies, while the organic fractions are used for breeding pigs [137]; in Dhanbad Municipality (India), informal recyclers play an important role in the plastic waste management, collecting the recyclable plastic waste from landfills, rendering environmental and social benefits [138]; In Bogotá (Colombia), informal recyclers collect materials from waste, motivated by profits, due to the free-market enterprise for recycling [136]; in Nuevo Laredo (México), where migration has increased the population to over 250,000 inhabitants, unemployed informal recyclers recovered 20 kg of aluminum cans and cardboard per day, making in one day the minimum-wage of one week of a factory worker [139]. In all these international realities, the main factors that allows the activity of the informal sector is the presence of low-income communities, unemployment, lack of MSW collection and the free management of waste.

Therefore, the activity of the informal sector contributes directly to the recovery of the materials and the reduction of environmental contamination. This practice is in accordance with the circular economy (CE) principles. The objective of the CE is closing of material loops, to prevent waste from final disposal, and transforming the resulting residual streams into new secondary resources [140]. It proposes a system where 4Rs provide alternatives to the use of raw virgin materials, making sustainability more likely [141]. The CE typically includes economic processes such as “reverse logistics” or “take back” programs that recover wastes for beneficial reuse, avoiding final disposal costs, often reducing raw material costs and even generating incomes [142]. Therefore, the inclusion of the informal sector represents a key strategy for improving the CE concepts, improving social, environmental and economic sustainability [143].

The activities of the informal sector regard the degree of formalization, from unorganized individuals in dumpsites, to well organized cooperatives. Therefore, issues such as exploitation by middlemen, child labor and high occupational health risks need to be challenged for addressing sustainability [144]. Globally, SWM remains a negative economy, where individual citizens pay the cost, the financial viability of recycling is disputed, and the sector remains vulnerable to great price volatility. Most of the collection systems in developed countries are subsidized, and also result in substantial exports of recyclables in global secondary resources supply chains. Moreover, if taxes, health insurance, child schooling and training provisions, management costs and other typical costs are included within the informal waste sector, it is not clear if the sector come back to being unsustainable economically [144].

It is recognized that a door-to-door collection service of source-separated recyclables may be one of the best solutions for improving RR. Therefore, the informal sector has the opportunity to deliver important environmental benefits, becoming an active agent of behavior change. Moreover, its activity can reduce the waste inflow into water bodies, decreasing the amount of marine litter in the oceans. The inclusion of the informal recycling should be more investigated, assessing pros and cons of its activity in different realities worldwide [144].

## 5. Discussion

From the review, it is clear that there is a strong linkage between poor SWM and environmental/health issues. The rapid increase in population, economic growth, urbanization and industrialization improve the generation of SW at global level, boosting environmental contamination when such SW is not managed. Indeed, in many developing countries waste is scattered in urban centers or disposed of in open dump sites. The lack of infrastructure for collection, transportation, treatment and final disposal, management planning, financial resources, know-how and public attitude reduces the chances of improvement, as pointed out also by other authors [145]. In Table 4 the main source of contamination and health risks due to SW mismanagement for each waste stream are summed up.

Nevertheless, the generation of SW can be also considered a source of opportunities: generation of renewable energy, new employment, new economic advantages, private investments and improvement of population awareness about environmental issues. In developing countries, the informal sector plays the main role in recycling where plastic, glass, metal and paper have a developed market. Appropriate strategies should be introduced for supporting these activities, such as improved public awareness, enaction of specific laws and regulations and implementation of SWM infrastructures. For instance, a study conducted in Bogotá (Colombia), found that the main external requirements for including the activity of the informal sector regards the recognition of recyclers’ work, the formal alliances with the productive sector and the stabilization of the prices of recycling material [146]. Therefore, actions should be implemented both by private companies and local governments.

Support can be provided with the assistance of NGOs, private companies or international funds, for boosting the 4Rs, which included waste separation at the source involving residents, institutions, local governments and local companies. A good example was provided in Managua, Nicaragua, where over the last five years, several international cooperation projects have focused on the improvement of SWM systems creating multi-stakeholder platforms, designing and implementing joint activities for improving technical capacity and awareness, boosting the implementation of integrated and appropriated projects [147]. Therefore, some recommendations should be introduced for improving the SWM systems at global level, as also suggested by other authors [148]:

- Improve public education and awareness among citizens and waste pickers,
- Improve financial sustainability of the SWM systems,
- Involve several stakeholders for improving system resilience,
- Include safety precautions in the informal recycling sector,
- Implement studies for assessing waste composition and characteristics.

In developing countries, in agreement with the results of a LCA study, good environmental protection can be accomplished by recycling and composting, since high amounts of organic fraction MSW are associated with environmental impacts [102], while inclusion of the informal sector is suggested due to the low economic investment required and technological simplicity [149]. Such options are in agreement with the circular economy (CE) principle, an emerging topic that has attracted research interest. However, three components should be included in the definition of CE [150]: re-circulation of resources and energy, recovering value from waste; implementation of multi-level approach; assessing the innovation introduced within the society. These principles are mainly implemented in European countries and in China, while in low income countries these activities are still under development [151]. Furthermore, another study found that the main incentive for the development of SWM in municipalities was the economy; the environment and public health are only secondary drivers [152]. CE patterns specific for developing countries should be introduced, focusing on big cities, since financial sustainability, multi-level approaches, and energy recovery are options that to date are not affordable in these contexts. Therefore, the scientific literature and research should move to this direction, providing sustainable solutions for low-middle income countries and appropriate technologies for boosting the CE.

## 6. Conclusions

The article presented a narrative review about environmental contamination and social issues in developing countries due to SW mismanagement. Results show that the SWM system should be considered in an integrated manner in order to cope with the reduction of the environmental footprint and to improve the targets of the SDSs. Too many times, SWM is considered as a single stream disposed in open dump sites. However, the implementation of future management plans requires the application of ad hoc collection and treatment solutions for each waste flow produced in municipal areas: MSW (organic and inorganic), HW, C&D waste, WEEE and used batteries, industrial and hazardous waste and used tires. Stakeholders and governments should know that SWM is a complex system that involves environmental, social and economic issues, which should be evaluated holistically for improving the life cycle of waste, reducing water, soil and air contamination due to open burning and open dumping, practices widespread worldwide.

Inclusion of the informal sector can be considered a viable way for improving the recycling rate and reducing the waste inflow into final disposal sites in developing countries, due to low technological requirements and economic investments. However, further investigations and efforts should be implemented for understanding the most appropriate strategy for its involvement. In Latin America various pilot project were implemented by the organization of cooperatives including waste pickers that have provided good results. However, in some areas of Asia and Africa this practice is forbidden and represents an obstacle to a formal selective collection system. Therefore, specific patterns should be implemented for each context, exploiting the activities just in place introducing the CE principles, remembering that informal recycling cannot be the only system in action; improving waste collection and selective collection coverage of municipal areas, introducing awareness and information campaigns, implementing appropriate treatment systems with regulations and control agencies, improving final disposal sites and its management, enhancing financial sustainability of the systems and introducing future management plans are all practices required for improving the integrated SWM system of a country, region, municipality or rural area.

From this review it is clear that common projects should be introduced at a global level in order to reduce the environmental contamination and health issues due to waste open dumping and burning. Authorities and the actors involved in waste management should be aware of the global issues which are affecting sustainable development, providing such information to the population for spreading awareness and its inclusion in recycling and prevention activities, also available within the scientific literature and this review. It should be specified that waste mismanagement has impacts at three levels: municipal or local impacts, such as soil and groundwater pollution, spread of diseases due to animal vectors (mosquitos, rodents) and air contamination; regional impacts, due to pollution of waterbodies used for agriculture and household purposes; global impacts, such as global warming and marine littering. Therefore, a common front should be organized for reducing these impacts globally, for improving environmental conditions and sustainable development.

## Figures and Tables

**Table 1:** Contaminants’ concentration in soil, runoff and groundwater due to open dumping in eight case studies, compared with international standard of soil contamination limits and drinking water.

Ref.	City/Region	Country	Environment Polluted	Pollutant	Concentrations	Limits
[48]	Chennai city	India	Soil (mg kg^−1^)	Zn	0.27–0.48	50
				Cu	3.78–0.59	100
				Fe		
[50]	Tiruchirappalli	India	Soil (mg kg^−1^)	Mn	171.16	500
				Pb	291.3>	50
				Cd	47.7>	4
[51]	Havana	Cuba	Soil (mg kg^−1^)	Cobalt	8.4	20
				Ni	50>	30
				Cu	252>	100
				Zn	489>	50
				Pb	276>	50
[52]	Uyo	Nigeria	Soil (mg kg^−1^)	Pb	9.9–11.8	50
				Zn	137–146>	50
				Ni	11.8–12.6	30
				Cr	3.6–4.1>	1
				Cd	9.05–12.2>	4
				Mn	91.2–94	500
[49]	Nonthaburi	Thailand	Runoff (mg L^−1^)	Mn	0.49>	0.4
				Cr	0.99>	0.05
				Cd	0.01>	0.003
				Pb	0.1>	0.01
				Ni	0.5>	0.07
				Zn	1.32	4
				Cu	0.63	2
				Hg	0.95>	0.002
[50]	Tiruchirappalli	India	Groundwater (mg L^−1^)	Cd	0.16–1.04>	0.003
				Cu	0.6–2.7	2
				Mn	0.2–1.8>	0.4
				Pb	0.8–5.1>	0.01
[53]	Mexicali	Mexico	Groundwater (mg L^−1^)	BOD_5_	4.3–6.5	20 *
				COD	23.5–188>	120 *
				Na	600>	200
				SO_4_^-^	1000>	300
[54]	Sepang	Malaysia	Groundwater (mg L^−1^)	BOD_5_	128–142>	120
				COD	2698–2891>	120
				Cl	123.8–127.7>	5
				Ni	0.44–0.65>	0.07
				As	0.06–0.07>	0.01
				Pb	0.04–0.08>	0.01
[55]	Alexandria(Landfill)	Egypt	Groundwater (mg L^−1^)	Ni	0.007–0.152	0.07
				Pb	0.002–0.009	0.01
				Cr	0.006–0.058>	0.05
				Mn	0.039–0.673>	0.4
				Cd	0.001–0.051>	0.003
				Zn	0.001–0.343	4

Note: Soil contamination limits [56], Drinking water limits [57], * water release after wastewater treatment.

**Table 2:** Categories of HW as reported by the WHO [79].

Waste Category	Description and Examples
Sharp waste	Used or unused sharps (e.g., needles, syringes with attached needles, knives, blades, broken glass).
Infectious waste	Waste suspected to contain pathogens and that poses a risk of disease transmission (e.g., waste contaminated with blood and other body fluids).
Pathological waste	Human tissues, organs or fluids, body parts, fetuses, unused blood products.
Pharmaceutical waste	Pharmaceuticals that are expired or no longer needed.
Chemical waste	Waste containing chemical substances (e.g., laboratory reagents, film developer, disinfectants that are expired or no longer needed, broken thermometers with mercury).
Radioactive waste	Waste containing radioactive substances (e.g., unused liquids from radiotherapy or laboratory studies).
Non-hazardous or general HW	Waste that does not pose any biological, chemical, radioactive or physical hazard.

**Table 3:** Comparison of the waste pickers’ activity among seven different countries worldwide.

Ref.	City	Country	No. of Waste Pickers	Organization/ Formalization	Source of Recyclables	Kg d^−1^ Per Waste Picker	Waste Fractions	Issues
[134]	Kathmandu	Nepal	7000–15,000	No	City streets/ landfill	60	Plastic bottles, plastic bags, paper, glass, iron, HW	Illnesses, lack of financial resilience, occupational risks
[130]	Balantyre	Malawi	N.A.	No	Open dumps in urban areas	20–30	PET, HDPE, LDPE, metals	Negative public perception, lack of capital, fluctuation of the price
[131]	Harare	Zimbawe	220	Licensed	Open dumps	70	Plastic, paper, rubber, metals, glass, tires	Competition with others waste pickers safety issues, discrimination, climate conditions
[129]	Ulaanbaatar	Mongolia	5000–7000	No	Dumpsites, public areas, streets	N.A.	Plastics, cans	Alcohol addiction, no ID card, homeless, discrimination, diseases
[128]	Beijing	China	150,000	Prohibited by regulation	Households, public bins, small enterprises	14–16	WEEE, paper, metals, plastics	Minimum wage standards, discrimination
[135]	Great Accra region	Ghana	N.A.	No	Landfills, open dump sites	N.A.	Metals, plastics, PET, WEEE	Health hazards, cuts & injuries, insects bites, lack of respect, unstable prices
[136]	Bogotá	Colombia	20,000	Cooperatives	Trash bags, public bins	25	Plastics, metals, paper, glass	Lack of public acceptance, health, cleanness of operation.

Note: (No.) number, (N.A.) not available.

**Table 4:** Environmental and health risks due to waste open burning and open dumping for different waste streams.

Waste Stream	Pollutants and Hazards	Environmental and Health Risks
MSW open dumping	Leachates with high concentrations of heavy metals, BOD, COD, SO_4_^2−^, NH_3_, Anaerobic digestion of organic fractions with generation of landfill gases, mainly composed of methane, Disease vectors living in the areas.	The leachate generated is released to the soil, polluting groundwaters mainly used for drinking and household purposes. The risks concern the health the population through direct and indirect (agriculture) intake. The generation of methane and other GHGs increases global warming, the risk of local fires and the pollution of the atmosphere surrounding the final disposal sites. The breeding of animals around the disposal sites and the presence of rodents and insects increases the risks of diseases transferring to the population through bites and direct contact with the animals. The uncontrolled disposal causes the release of waste fractions, mainly plastics, into water bodies, contaminating the rivers, lakes and then the oceans and the seas, causing the phenomena of the marine littering.
MSW open burning	Generation of PCDD/F and cancerogenic compounds, PM, BC, CO, CO_2_, NO, and other GHG and hazardous compounds.	The emissions due to uncontrolled waste fires produce significant amounts of contaminants that affect the health of the population. Respiratory illnesses, especially in children, are common in areas with open burning practices. The generation of BC, CO, CO_2_ and other GHG, affects the GWP, more than the anaerobic degradation of organic waste.
HW	Open dumping of sharp and infectious waste, Burned HW generates PCDD/F and other hazardous compounds.	The presence of sharps and infectious waste in open dump sites increases the risks to waste pickers that operate in the area. Indeed, recyclable materials are scavenged by informal recyclers, that are not aware of the issues due to HW. Moreover, these fractions can be targets for animal disease vectors. Finally, the open dumping of HW creates bacterial resistance, that affects the performance of antibiotics for human uses. Burned HW is a source of PCDD/F that directly affect the health of the population living near sites, increasing the risk of cancer and respiratory diseases.
WEEE and used batteries	The open dumping of the waste generates leachates with high heavy metals concentrations, The open burning generates hazardous compounds like PCDD/F, BC and PM.	Open WEEE dumping generates leachates rich in heavy metals. Waste picking is also done on these fractions to collect precious metals. These practices affect the health of the pickers that operate in the dumping sites, due to the presence of Hg, Pb, Cd and Mn, all hazardous metals. Picking of WEEE leads to waste burning for the recovery of the metals. Uncontrolled WEEE burning affects the quality of the air and the atmosphere due to the generation of high amounts of PM, BC and PCDD/F, affecting both the health of the populations surrounding the site and increasing the GWP.
C&D waste	Landslides due to waste uncontrolled dumping, Presence of hazardous materials within the waste, such as asbestos, lead, mercury and sharp waste.	The main issue is the risk of landslides that can affect populated areas. The huge amounts of waste produced by C&D activities reduce the useful life of final disposal sites and their density, increasing the risk of land collapses. The presence of hazardous materials can be a source of pollution due to the leachates generated at the final disposal sites or directly for the waste pickers operating collecting recyclable materials. Pb and Hg waste can affect the health of the population due to respiratory, skin and other illnesses.
Waste tires	The open dumping causes the presence of mosquitos and the risk of fire injections, Open burning generates contaminants for the atmosphere, such as BC and SO_x_.	Open dumping of waste tires is an area of mosquito growth, especially in tropical areas, where dengue, malaria, yellow fever, among other diseases, is common. The presence of waste tires in open dump sites increases the risk of contraction of these illnesses. Moreover, the presence of this highly combustible waste fraction, can be a cause of fires. Open burning of waste tires induces high generation of PM, BC and SO, increasing the GWP and the acid rain phenomenon due to the presence of sulphates in the atmosphere which generate H_2_S in contact with water, increasing environmental pollution. Moreover, the generation of PM, containing heavy metals increases health issues in the populations that live near the areas, also affecting the air indoor.
Industrial waste	Generation of hazardous leachates mainly composed of heavy metals.	The presence of heavy metals affects the health of citizens, especially the children, which intake can be less than an adult in terms of concentrations assumed per day, boosting the health effects. The presence of heavy metals is persistent and affects the soil and groundwater quality, with possible intake by direct ingestion (e.g., food and water).
